# Flight crew fatigue risk assessment for international flights under the COVID-19 outbreak response exemption policy

**DOI:** 10.1186/s12889-022-14214-5

**Published:** 2022-10-01

**Authors:** Junya Sun, Ruishan Sun, Jingqiang Li, Ping Wang, Nan Zhang

**Affiliations:** College of Safety Science and Engineering, Civil Aviation University of China, Tianjin, 300300 China

**Keywords:** COVID-19, Exemption policy, Flight crew, Fatigue risk level, Model simulation, Empirical study

## Abstract

**Background:**

In response to the COVID-19 outbreak, the Civil Aviation Administration of China (CAAC) has formulated Implementation Measures for Exemption of Crew Duty Periods and Flight Time Restrictions during the COVID-19 Outbreak. This exemption policy imposes temporary deviations from the approved crew duty periods and flight time restrictions for some transport airlines and regulates the use of multiple crews for continuous round-trip flights. However, no research has been conducted on flight crew fatigue under this exemption policy. That is, the exemption policy lacks theoretical analysis and scientific validation.

**Methods:**

Firstly, flight plans for international flights under both the exemption and the CCAR-121 Policy schemes (with three flight departure scenarios: early morning, midday and evening) are designed, and flight plans are simulated based on the SAFE model. The Karolinska Sleepiness Scale (KSS) and the PVT objective test of alertness, both of which are commonly used in the aviation industry, are then selected for use in an empirical experimental study of flight crew fatigue on two flights subject to the exemption and CCAR-121 policies.

**Results:**

The SAFE model simulation found that the fatigue risk results based on flight crews for flights departing in the early morning (4:00), at noon (12:00) and in the evening (20:00) indicate that the fatigue risk levels of flight crews operating under the exemption policy are overwhelmingly lower than or similar to those operating under the CCAR-121 policy. However, there were a few periods when the fatigue risk of crews flying under the exemption policy was higher than that of crews flying under the CCAR-121 policy, but at these times, the crews flying under both policies were either at a lower level of fatigue risk or were in the rest phase of their shifts. In the experimental study section, 40 pilots from each of the early morning (4:00), noon (12:00) and evening (20:00) departures operating under the exemption policy were selected to collect KSS scale data and PVT test data during their duty periods, and a total of 120 other pilots operating under the CCAR-121 policy were selected for the same experiment. First, the KSS scale data results found that flight pilots, whether flying under the exemption policy or under the CCAR-121 policy, had overall similar KSS scores, maintained KSS scores below the fatigue risk threshold (i.e., KSS score < 6) during the flights and that the empirical KSS data and the model simulation results from the KSS data were overall identical at the test nodes during the flight and had nearly identical trends. Finally, the results of the PVT objective test indicators showed that the overall change in 1/RT of the crews flying under the exemption policy was less than or similar to that of the crews flying under the CCAR-121 policy, while the maximum change in 1/RT of the crews under both policies was between 1 and 1.5. This indicates that the overall level of alertness of the crew flying under the exemption policy is higher than or similar to that of the crew flying under the CCAR-121 policy, while the change in alertness level of the crew before and after the mission is relatively small when flying under either policy.

**Conclusion:**

Based on the model simulation results and the results of the empirical study, it was verified that the overall fatigue risk level of flight crews operating under the exemption policy is lower than or similar to the fatigue risk level of flight crews operating under the CCAR-121 policy. Therefore, the exemption policy in response to the COVID-19 outbreak does not result in an overall increase in the level of flight crew fatigue risk compared to the original CCAR-121 policy.

## Background

The global outbreak and spread of COVID-19 pose a threat to life and safety while also having a significant impact on the economic development of various industries in various countries [1]. The civil aviation industry, as an industry of a global nature, has a market that is closely linked to the changes in the global epidemic [2]. Therefore, to cope with the new and complex requirements of epidemic prevention and control, to protect the health of crew members and to meet the requirements of passenger and cargo transportation in emergency situations, the Civil Aviation Administration of China (CAAC) has formulated the Regulations on the Implementation of Exemptions from Duty Periods and Flight Time Restrictions for Crew Members during the Epidemic (hereinafter referred to as the “Exemption Measures”) [3]. The exemptions are based on the requirements of the Rules for the Operational Qualification of Carriers of Large Aircraft for Public Air Transport (China Civil Aviation Regulations-121, Part CCAR-121) [4] and impose temporary deviations from the crew duty periods and flight time limits for some transport airlines, further regulating the management of extended crew duty periods and flight times for the use of multiple crews on intercontinental routes. The exemption from the multiple crew round trip operational model allows for flight crew duty periods and flight times that exceed the limits of the original CCAR-121 regulations but increases the number of flight crews and improves rest facilities on board the aircraft, thereby reducing the amount of rest time, as no overnight stays at the destination are required. It is intended that this mode of operation will meet the requirements of passenger and cargo transportation in emergency situations while also reducing the risk of crew members contracting COVID-19, reducing crew working hours, mitigating the risk of crew fatigue and ensuring the safe and reliable operation of flights. However, there is a lack of theoretical analysis and scientific validation of the safety and risk of pilot fatigue associated with this mode of operation, which exceeds the limits of the previous regulations and attempts to safely increase crew flight time by increasing the number of people. In addition, due to the extended duty periods and flight times of crew members, as well as the rotational working pattern of multiple sets of crews onboard at the same time, how to predict and monitor the fatigue conditions of crew members and the change of alertness of flight crew in the cabin during the route operation has become the supervision focus of the bureau and the company.

Traditional methods for predicting changes in crew fatigue status and alertness are divided into subjective evaluation methods, such as questionnaires/scales, objective monitoring methods, such as physiological and behavioural performance indicators, and a predictive simulation method based on biomathematical models. In 2012, when the ICAO recommended that states establish data-driven, continuous monitoring and management of fatigue risk management systems (FRMSs) based on scientifically valid principles and measurements, it suggested that biomathematical models could be used to identify and predict fatigue risk for crew members [5–7]. Biomathematical modelling is currently a recognised scientific method that uses physiological parameters related to the organism as input data to create a series of mathematical models in the form of a system of equations [5, 8]. It integrates scientific research on human circadian rhythms, sleep, workload and alertness in relation to fatigue risk with flight production planning and scheduling and is able to visualise trends in fatigue during the planned duty period and predict the potential fatigue risk [5, 9]. Therefore, the biomathematical model of fatigue can assess and monitor the fatigue levels of crew members at all times during duty and flight and solve the problem of quantitative pilot fatigue measurement, which is of great importance for the preintervention of flight fatigue before the crew is scheduled for duty, the monitoring of crew members’ alertness in the cockpit, the evaluation of crew members’ fatigue statuses, and guaranteeing flight safety on the route.

This paper applies the Fatigue Scale, which is a subjective evaluation method, the Psychomotor Vigilance Task (PVT), which is an objective test method, and the SAFE model to detect and predict the risk of fatigue for flight planning under the flight time restrictions of the Exempted Approach Regulations and the CCAR-121 Part Regulations. The fatigue risk prediction results of the exemption approach and the CCAR-121 Part Regulations are compared, while the fatigue risk levels under both regulations are analysed with empirical data (scale data and PVT data) from real flight pilots, and the prediction results of the model are validated. Through a combination of model prediction and experimental validation, the exemption is found to be reasonable and safe; thus, the CAAC can meet the requirements of epidemic prevention and control and provide scientific support for fatigue monitoring and management.

Based on the purpose, content and methodology of this study, a number of hypotheses are proposed.

- Hypothesis 1: A flight crew contains a captain and copilot who are equally fatigued at work and at rest;
- Hypothesis 2: All take-off tasks have same effect on fatigue, all landing tasks have the same effect on fatigue and all cruising tasks have the same effect on fatigue;
- Hypothesis 3: At the time of the formal empirical experiment, the statuses of all pilots included in the experiment were similar;
- Hypothesis 4: The effect on pilot fatigue of a flight from Shanghai to Frankfurt is similar to that of a flight from Chongqing to Amsterdam in terms of time difference and flight time;
- Hypothesis 5: 4:00, 12:00 and 20:00 can represent the departure times of flights in the early morning, midday and evening, respectively.

## Comparison of the two policies

Part CCAR-121 (China Civil Aviation Regulations-121, Part CCAR-121) is a regulation issued by the Civil Aviation Administration of China (CAAC) for the purpose of conducting operational conformity certification and continuous supervision and inspection of large aircraft public air transport carriers to ensure that they meet and maintain the required operational safety level [4].

Exemptions, i.e., “Implementation of Exemptions from Crew Duty Period and Flight Time Limits during an Epidemic” (Exemptions), apply to the operation of certain large aircraft public air transport carriers proposing to use multiple crews for consecutive round-trip flights during an epidemic that exceed the crew duty period and flight time limits set by Part CCAR-121 regulations [3].

Table 1 compares the restrictions on crews in both the exemption approach and the CCAR-121 Part regulations and finds that the maximum flight time in the exemption approach is 8–13 h more than that in the CCAR-121 Part, with twice the number of crew members. That is, the exemption increases the number of flight crews while extending the flight time limit and introduces the requirement for rest facilities on board the aircraft, with to the aim of reducing the working hours of the crews, mitigating the risk of crew fatigue and ensuring the safe and reliable operation of flights.

**Table 1:** Restrictions on crews in the exemption and CCAR-121 policies

Policy	Number of crew members/person	Maximum flight duty period/h	Maximum flight time limit/h	Transit break arrangements
CCAR-121^a^	3	16–18	13	Receive a rest period of at least 10 consecutive hours
	4	18–20	17	
Exemption options^b^	6	30	26	A ground rest period of at least 3 consecutive hours and in a rest environment that meets the requirements of a Level 2 rest facility, and the rest period is not counted as part of the flight duty period
		26	21	
	8	35	30	
		26	21	

^a^, the CCAR-121 Part expansion flight crew should include at least one person qualified as a captain and one person qualified as a cruise pilot or above. ^b^, there should be at least three or more complete flight crews, each of which should consist of at least one qualified captain (including a cruise captain) and one qualified copilot

## Research methodology

### SAFE model simulation calculation

The SAFE model is a composite biomathematical fatigue prediction model designed for civilian pilots that was originally a neuroscience and sleep research project to help the UK Ministry of Defence understand the degradation of pilot performance when flying for nine consecutive days. The developers therefore based the results on continuous research since the early 1980s, including the identification and measurement of possible pilot fatigue on tens of thousands of schedules. The UK CAA then went on to support a more detailed study of pilots from many airlines around the world, creating the SAFE biomathematical model and using it as part of the evidence to approve (or not) the repeal of a regulation. It is designed for aviation applications and has been validated specifically for use in the aviation industry on behalf of the UK CAA, as it has collected a wealth of data from over three decades of crew missions conducted by commercial air carriers worldwide [10–15].

The SAFE model inputs. The SAFE model has been validated with extensive pilot sleep data and fatigue monitoring data. In addition, the format of the data is less demanding; Table 2 illustrates the input data reference template for the SAFE model.

**Table 2:** SAFE model input parameter data information

Duty	Airport	Sleep	Rest	Other information
On date+time; Off date+time	Start; End; Home base	Count; Order; class	First/second start date+time; First/second start date+time	Crew composition; Time zone

The SAFE model output generates predicted SP fatigue scores (on a 7-point scale) at 15-minute intervals throughout the work period, as well as other scale scores, such as KSS fatigue scores (on a 9-point scale), and predicts how sleep is likely to occur within them. The model also shows the progression of fatigue and predictions of sleep during each task of the schedule in gradient coloured bars, as shown in Table 3, Fig. 2, etc.

**Table 3:**
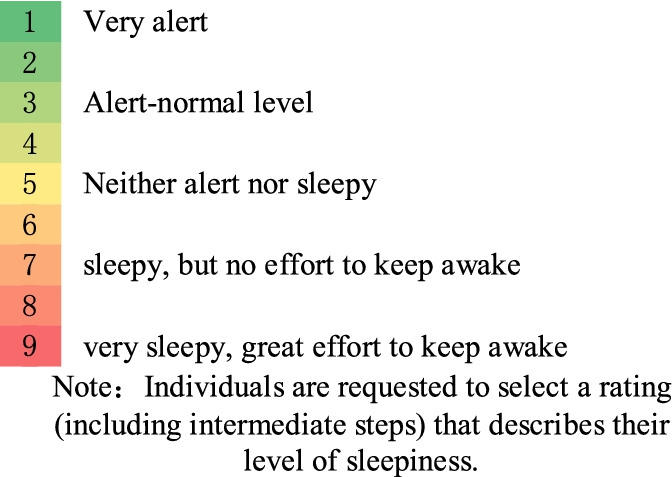
The Karolinska Sleepiness Scale (KSS)

Individuals are requested to select a rating (including intermediate steps) that describes their level of sleepiness

It is particularly important to note that the mechanical models are based on data from shift workers and are therefore limited in the length of work in their datasets, while the empirical models face the same limitations, but SAFE has been validated for extra-long working hours of up to 22 hours. In addition, the SAFE model has helped many airlines predict the fatigue that can occur during missions of up to 36 hours, so SAFE can be extrapolated to a maximum of 30 hours and still provide a good estimate of fatigue [5, 16, 17]. China’s proposed civil aviation exemption policy in response to the COVID-19 outbreak exceeds the original CCAR-121 policy limit of a 35-hour maximum flight duty period. Therefore, the model is applied to assess the prediction of crew fatigue in the scheduling table for the exemption policy.

With the development of biomathematics, the current biomathematical models of fatigue include the two-process model of sleep regulation (TPMSR) [18], the three-process model of alertness (TPMA) [19], sleep activity fatigue and task effectiveness (SAFTE) [8], fatigue audit interDyne (FAID) [20], the circadian alertness simulator (CAS) [9], the sleep/wake predictor SWP [21] and the interactive neurobehavioral model (INM) [22], among others. However, most of the above models are mechanistic models, which are built by analysing the three processes of sleep homeostasis (waking time), circadian rhythm and sleep inertia. These models do not distinguish between work and nonwork activities and are essentially concerned with sleep deprivation [10]. The sleep deprivation model does not take into account the number of segments flown, nor does it take into account the number of passengers (for the crew) who are unruly due to the so-called “hassle factor”, unpredictable bad weather, type of airport, high air traffic control interventions, newly qualified pilots who are not fully familiar with their schedules, poor lighting, etc. This means that mechanical models will always underestimate the level of fatigue of the crew. In contrast, the system for aircrew fatigue evaluation (SAFE) [10–15], commissioned by the UK Civil Aviation Authority and designed, built and validated by the research team at FRMSc, the UK Ministry of Defence research agency DERA (now QinetiQ plc), is an empirical model based on empirical data, and empirical models perform better than mechanical models based on simulation studies of datasets. The SAFE model was built from the results of a continuous research programme in operation since the early 1980s. This vast research database contains tens of thousands of analysed schedules to identify and measure the causes of pilot fatigue and is by far the most studied biomathematical fatigue prediction model applied to civil aviation pilots (the original data collection came from many airlines such as British Airways, Air New Zealand and Japan Airlines), and in 2001, the SAFE model was the first biomathematical model of fatigue to be endorsed and recommended for use by official organisations such as EASA and the Civil Aviation Authority of Singapore. The SAFE model predicts the development of fatigue during tasks by generating predicted Samn Perelli (SP) and KSS scores (supporting optional other fatigue/alertness scales) every 15 minutes throughout the work period based on the input schedule. The SAFE model can therefore assess the risks associated with civil aviation-specific mission schedules, predict the levels of fatigue experienced by crews during a given mission schedule and thus propose corresponding changes to the regulations (this is the main reason we selected the SAFE model to validate the CAA exemption approach).

### Empirical studies

#### Subjects

The pilot group of a Chinese airline is selected for the empirical experiment. The empirical study is supported by Chinese regulators, airline unions and management, and the pilot group participating in the experiment signed a written informed consent form. From these, similar flights under two regulatory operating policies, i.e., Part CCAR-121 and the exemption approach, and 80 pilots from each of the three early, mid and late departure times, i.e., departing at approximately 04:00, 12:00 and 20:00, with ages ranging from 23 to 50 years old, are selected to extract data for analysis.

#### Experimental indicators

The International Air Transport Association (IATA) and the International Civil Aviation Organisation (ICAO) have identified three basic requirements for flight fatigue testing for airlines conducting FRMS [6]. The test must meet the following requirements: 1) the test method for pilot fatigue must be scientifically proven; 2) it must not impair the pilot’s ability to perform the task; and 3) it must be widely used in the aviation field. Based on these recommendations, we selected the Psychomotor Vigilance Task (PVT) test tool, a fatigue detection technique based on visual reaction time that has been widely used in studies related to sleep, cognition and fatigue and has been suggested by the aviation industry as an objective measurement tool for flight crew fatigue assessment [23]. For example, NASA has launched the PVT Aviation study version of the NASA PVT+ APP testing tool for use in research experiments. The metrics obtained from PVT measurements include the mean reaction time, inverse reaction time (1/RT), fastest 10% reaction time, slowest 10% reaction time, standard deviation of mean reaction time, number of misses (reaction time ≥ 500 ms), probability of misses (number of misses divided by the number of valid stimuli), and number of prejudgments (reaction time < 100 ms). The changes in the different indicators allow the evaluation of the fatigue state of pilots in different dimensions of physiology and psychology [23–26]. From the range of performance metrics generated by the PVT test, we selected the metric 1/RT for evaluation (reason for selection: in a summary of 141 journal papers published between 1986 and 2010 reporting PVT results, it was found that the most commonly used test metric in terms of the use of PVT outcome metrics was the number of misses, with a frequency of 66.7%; mean RT was 40.4%; and mean 1/RT was 30.5% [27].; in addition, the study analysed the indicative effects of different PVT outcome indicators and found that the number of omissions yielded high effect values, but the mean 1/RT had a somewhat higher effect value and was therefore recommended as the main outcome metric parameter indicator). In addition, we chose a PVT test length of 300 seconds or five minutes (reasons: determined by both the time limit required for PVT testing and the need to avoid excessive interference with mission requirements, and the fact that a 5-minute PVT test has been shown to be effective in indicating changes in alertness [28], i.e., for some performance indicators, a 5-minute PVT test can be used instead in applications where a 10-minute PVT is not appropriate) test length in time-limited work environments (e.g., aircraft cockpits, air traffic control rooms). The PVT can be applied at intervals of 10 seconds between the end of each stimulation time. Many fatigue detection scales exist, of which the KSS scale is one of the more used fatigue scales in the aviation industry [29]. In addition, the SAFE model also provides KSS scale scores, so we selected the KSS scale as one of the tools for crew fatigue measurement under both the exemption and CCAR-121 policies.

In addition, the PVT test software tool provides a subjective scale data collection tool, the Karolinska Sleepiness Scale (KSS), which is a sleepiness-based scale that uses a scale of 1 to 9; the higher the participant’s self-assessment score is, the more pronounced the subjective fatigue [30]. The KSS scale is also identical to the KSS scale given in the SAFE model, which provides good validation of the model’s predictions.

#### Experimental procedure

**Fig. 1:**
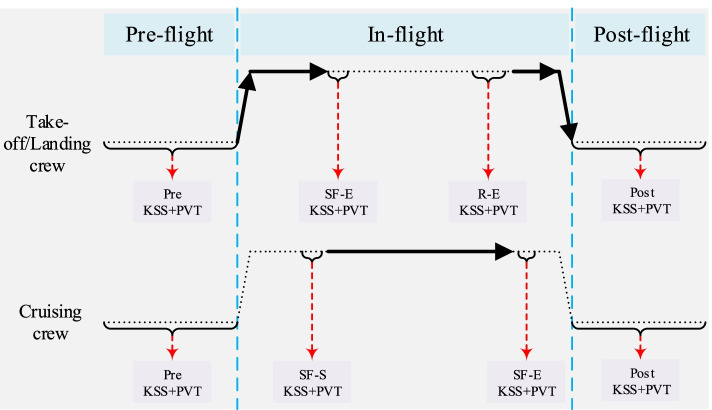
Flow chart for testing flight crews. Note: Pre, before the flight; R-E, end of in-flight rest; Post, end of flight; SF-S, start of flight shift duty; SF-E, end of flight shift duty

Figure 1 shows the flow chart for testing flight crews, and Table 4 describes the test flow during the flight task (combining Fig. 1 and Table 4 to interpret the experimental test process). The test flow for the subjects in question for the PVT is as follows:

- Step 1: On the day the assignment begins (preferably when you wake up, before duty), click on DUTY DAY, then WAKING UP, fill in one of the SLEEP DIARY and then perform the PVT test;
- Step 2: After the start of duty and before the flight begins, click on WORK TASKS, click on PREFLIGHT to fill in the KSS scale and then perform the PVT test;
- Step 3: Click on INFLIGHT to fill in the KSS scale and finally perform the PVT test when the test time points during the flight are designed according to Fig. 1;
- Step 4: After the aircraft has landed, click on POST DUTY, fill in the KSS scale, perform the PVT test, then click on BEFORE SLEEPING and fill in the sleep diary, and finally, the software will automatically exit;
- step 5: Before returning, click on DUTY DAY, then go to WORK TASKS, then click on PREFLIGHT and repeat steps 2 and 3 above;
- Step 6: After the final return landing, click on POST DUTY, fill in the KSS scale, perform the PVT test, click on BEFORE SLEEPING and fill in the sleep diary. Finally, the software will automatically exit.

**Table 4:** Crew testers and test moment design

Crew type	Test moment (one way)	
	Flight crew for each phase	All flight crew
Take-off crew^a^	Measured within 20 minutes of take-off and end of shift duties (start of in-flight rest); Measured within 20 minutes prior to taking over again.	Measured 2 hours prior to take-off; Measured before leaving the aircraft.
Cruising crew^b^	Measured within 20 minutes prior to the start of the shift; Measured within 20 minutes of the end of the current shift assignment.	
Landing crew^a^	Measured within 20 minutes prior to shift start;	

^a^Usually, one way take-off/landing with the same crew according to the internal rotation arrangement; ^b^ usually one way according to the design arrangement of 1 rotation

The above experimental procedure refers to the data collection procedure of Gander et al. [31]. However, since pilots are not allowed to perform activities other than flight work during take-off to cruise altitude (TOC) (for flight safety reasons), we chose the “SF-E, end of flight shift duty” phase to collect pilot data, as shown in Fig. 1, which is also in line with ICAO recommendations [5]. Similarly, the other test periods for our experimental study were determined in conjunction with the ICAO recommendation of “phases of flight that require focused monitoring of pilot status” [5].

### Flight work plan design under both the exemption and part CCAR-121 regulations

The flight work plan is first designed for an airline under both the exemption and CCAR-121 regulations, and then the SAFE model is applied to assess the fatigue risk of the flight work plan under both regulations.

For flights operated under the exemption approach, a flight work plan is designed based on the real mission flow of an airline for three early, mid and late departure times under the exemption approach operation at 04:00, 12:00 and 20:00, as shown in Table 5. Similarly, flight work plans are designed for three early, mid and late departure times under Part CCAR-121 operations at 04:00, 12:00 and 20:00 based on the company’s pre-epidemic operational requirements with reference to real flight plans, as shown in Table 6. Tables 5 and 6 are entered into the SAFE model.

**Table 5:** Flight work plan for a particular flight under the exempted approach operation (all times in Beijing time)

Flight start time	Crew	10/18 4:00–10/18 7:00	10/18 7:00–10/18 13:00	10/18 13:00–10/18 15:30	3 hours rest	10/18 18:30–10/18 21:30	10/18 21:30–10/19 3:00	10/19 3:00–10/19 5:00
Early morning	a	Flying	Resting	Flying	Resting	Resting	Resting	Resting
	b	Resting	Flying	Resting	Resting	Resting	Resting	Resting
	c	Resting	Resting	Resting	Resting	Flying	Resting	Flying
	d	Resting	Resting	Resting	Resting	Resting	Flying	Resting
Flight start time	Crew	10/18 12:00–10/18 15:00	10/18 15:00–10/18 21:00	10/18 21:00–10/18 23:30	3 hours rest	10/19 2:30–10/19 5:30	10/19 5:30–10/19 11:00	10/19 11:00–10/19 13:00
Noon	e	Flying	Resting	Flying	Resting	Resting	Resting	Resting
	f	Resting	Flying	Resting	Resting	Resting	Resting	Resting
	g	Resting	Resting	Resting	Resting	Flying	Resting	Flying
	h	Resting	Resting	Resting	Resting	Resting	Flying	Resting
Flight start time	Crew	10/18 20:00–10/18 23:00	10/18 23:00–10/19 5:00	10/19 5:00–10/19 7:30	3 hours rest	10/19 10:30–10/19 13:30	10/19 13:30–10/19 19:00	10/19 19:00–10/19 21:00
Evening	i	Flying	Resting	Flying	Resting	Resting	Resting	Resting
	j	Resting	Flying	Resting	Resting	Resting	Resting	Resting
	k	Resting	Resting	Resting	Resting	Flying	Resting	Flying
	l	Resting	Resting	Resting	Resting	Resting	Flying	Resting
		Outbound			Destination	Return trip		

a,b,c,d,e,f,g,h,i,j,k and l are all 2 pilots (pilot and copilot)

**Table 6:** Flight work plan of a flight under the operation of CCAR-121 (all times in Beijing time)

Flight start time	Crew	10/15 4:00–10/15 7:00	10/15 7:00–10/15 12:30	10/15 12:30–10/15 14:35	10 hours rest	10/16 0:35–10/16 3:35	10/16 3:35–10/16 8:35	10/16 8:35–10/16 10:35
Early morning	m	Flying	Resting	Flying	Resting	Flying	Resting	Flying
	n	Resting	Flying	Resting	Resting	Resting	Flying	Resting
Flight start time	Crew	10/15 12:00–10/15 15:00	10/15 15:00–10/15 20:30	10/15 20:30–10/15 22:35	10 hours rest	10/16 8:35–10/16 11:35	10/16 11:35–10/16 16:35	10/16 16:35–10/16 18:35
Noon	o	Flying	Resting	Flying	Resting	Flying	Resting	Flying
	p	Resting	Flying	Resting	Resting	Resting	Flying	Resting
Flight start time	Crew	10/15 20:00–10/15 23:00	10/15 23:00–10/16 4:30	10/16 4:30–10/16 6:35	10 hours rest	10/16 16:35–10/16 19:35	10/16 19:35–10/17 0:35	10/17 0:35–10/17 2:35
Evening	q	Flying	Resting	Flying	Resting	Flying	Resting	Flying
	r	Resting	Flying	Resting	Resting	Resting	Flying	Resting
		Outbound			Destination	Return trip		

m,n,o,p,q and r are all 2 pilots (pilot and copilot)

While the SAFE model has not yet begun to be validated for the 6/8 pilots flying round-trip flights in the CAA’s immunity exemption approach, it has been confirmed that the SAFE model’s validation dataset fully covers normal ultralong missions lasting up to 22 hours, while fatigue prediction for 30-hour missions performs equally well, with ultrahigh 30 hours requiring caution. Therefore, we first limited the flight schedule to 22 hours under the selected exemption approach operation, as shown in Table 2 (e.g., Pudong International Airport, Shanghai, China (PVG)-Frankfurt International Airport, Germany (FRA)), and we designed two sets of four people (two Groups A and B) to fly the outbound journey and another two sets of four people (two Groups C and D) to fly the return journey, which are treated as two separate flights for model simulation prediction. A flight schedule of 20 hours and 35 minutes (e.g., Jiangbei International Airport, Chongqing, China (CKG) - Amsterdam International Airport, The Netherlands (AMS)) under Part CCAR-121 operations similar to those listed in Table 2 is selected for the comparative validation of the exemption approach flights.

### Data processing methods

The above flight work plan is entered into the SAFE model for simulation, and the change in crew fatigue risk indicator KSS scale scores with duty time during each flight are analysed, as well as the corresponding model KSS scale score data selected according to the empirical study test time points (as shown in Fig. 1 and Table 4). The KSS data and PVT data for the pilot groups tested in the empirical study are selected according to the flight schedules described above and are selected separately, as shown in Fig. 1, to make a box line plot for each test time point. In addition, the KSS data selected by the SAFE model are validated against the test time node KSS data and PVT data, where the PVT data are selected as a 1/RT indicator.

## Results

### SAFE model prediction results

**Fig. 2:**
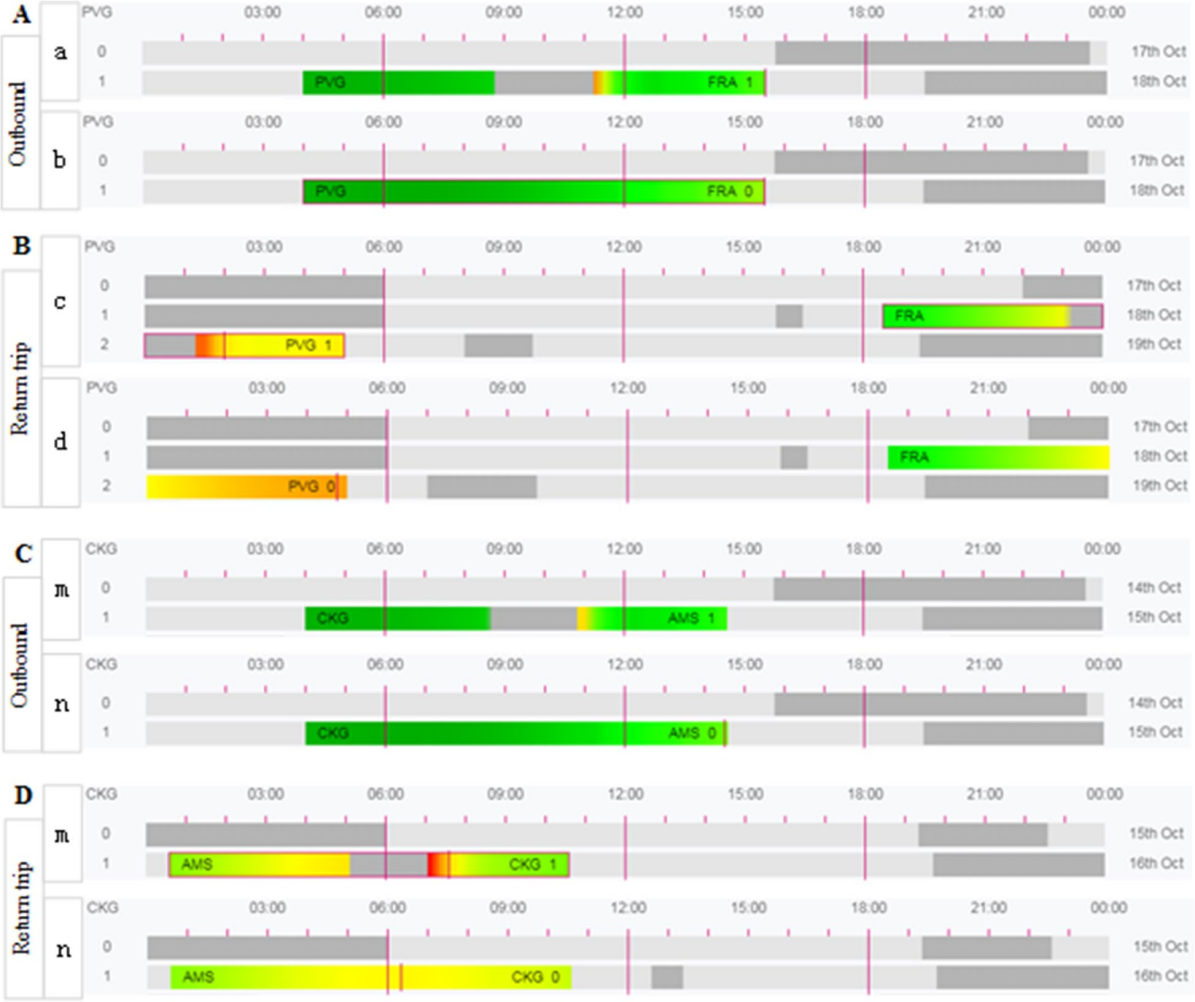
SAFE model simulation predictions for the 4 am flight schedule under the exemption and the CCAR-121 policies, with **A** and **B** showing the outbound (manned by flight crews a and b) and return (manned by flight crews c and d), respectively, and **C** and **D** showing the outbound and return (both manned by flight crews m and n). A comparison of **A** and **C** shows the fatigue risk for flight crews flying under the exemption approach and CCAR-121 regulations on outbound flights. A comparison of **B** and **D** shows a comparison of the fatigue risk for flight crews flying under the exemption approach and CCAR-121 regulations on return trip flights. Note: The meaning of the level of risk represented by the different colours is determined by Table 3. PVG stands for Shanghai Pudong International Airport; FRA stands for Frankfurt International Airport; CKG stands for Chongqing Jiangbei International Airport; AMS stands for Amsterdam International Airport. The number at the end of the duty refers to the number of segments flown during the time on duty

The latest SAFE model provided by FRMSc is selected and inputted into the above flight work plan designed in accordance with the exemption policy and the policy provisions of the CCAR-121 policy, and model simulations are carried out with the following results:

As shown in Fig. 2, with the exclusion of short periods of low red alert due to sleep inertia, the simulations show that the fatigue risks for the flight crew are similar and low for both policies when going downrange, with the same level of risk and neither reaching the red alert level (as shown by the comparison between Fig. 2A and C). A comparison of the results in Fig. 2B and D shows that the overall fatigue risk for the flight crew on the return trip under the exemption policy is higher than that under the CCAR-121 policy, particularly during the landing phase of the flight (shown in Fig. 2B-c and D-m), but that the KSS scores at this time are all intermediate (yellow) and therefore are not part of the higher fatigue risk phase. Figure 2B-d is in a rest phase when not undertaking a flight, although it has a red warning fatigue level during the landing phase of the flight (as observed from Tables 5 and 6). Therefore, as observed from Fig. 2, the levels of fatigue risk for flight crews under the exemption policy for departures in the early morning hours are either similar to the CCAR-121 policy (Fig. 2A and C) or higher than the CCAR-121 policy (Fig. 2B and D), but all are at an intermediate level of fatigue risk, with flight crews in the higher fatigue risk hours not undertaking flight duties.

**Fig. 3:**
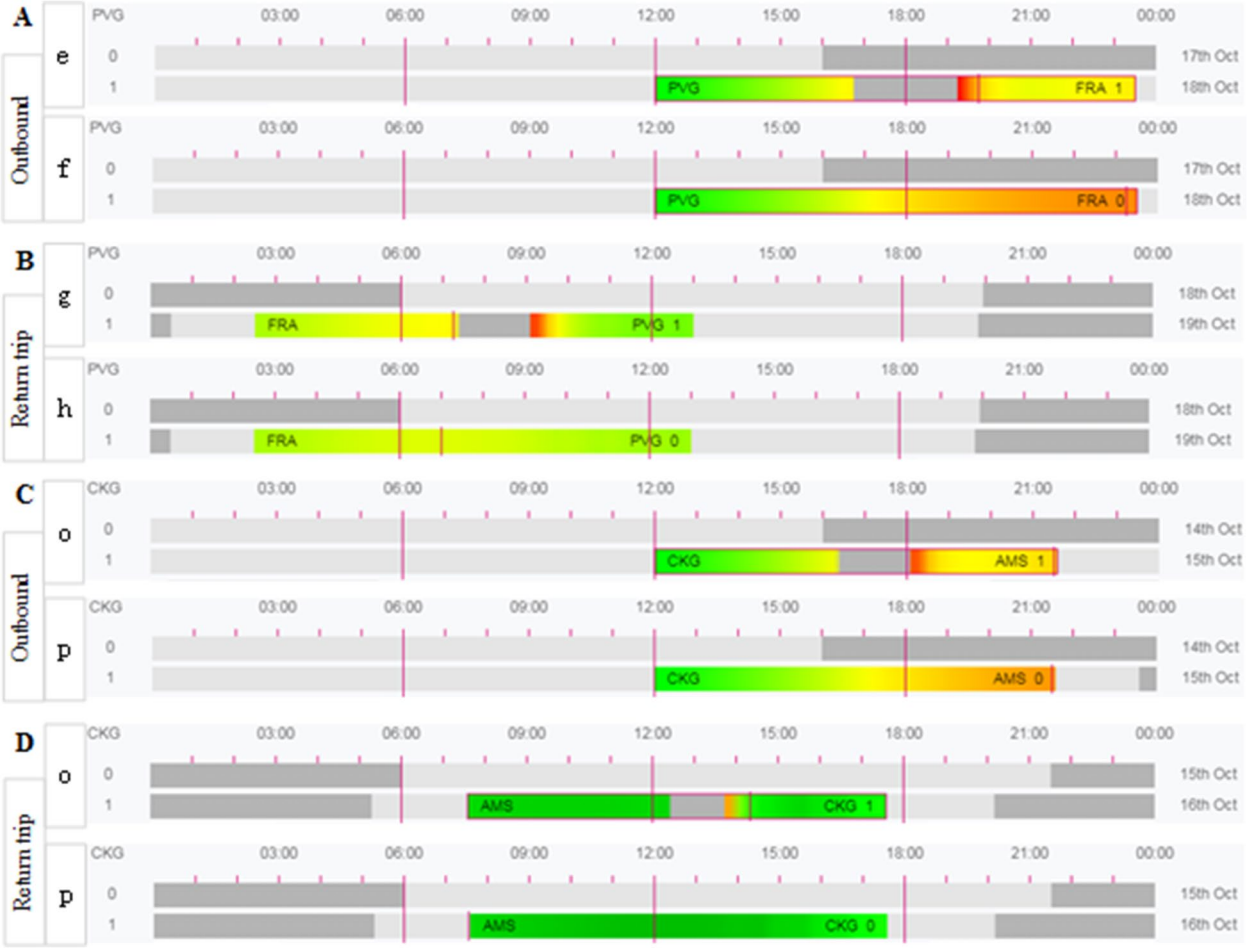
SAFE model simulation predictions for the 12:00 noon flight schedule under the exemption and the CCAR-121 policies, with **A** and **B** showing the outbound (manned by flight crews e and f) and return (manned by flight crews g and h) flights, respectively, and **C** and **D** showing the outbound and return (both manned by flight crews o and p) flights, respectively, under the CCAR-121 policy. Crews o and p are on duty). A comparison of **A** and **C** shows the fatigue risk levels for flight crews flying under the exemption approach and CCAR-121 regulations on outbound flights. A comparison of **B** and **D** shows a comparison of the fatigue risk levels for flight crews flying under the exemption approach and CCAR-121 regulations on return trip flights. Note: The meaning of the level of risk represented by the different colours is determined by Table 3. PVG stands for Shanghai Pudong International Airport; FRA stands for Frankfurt International Airport; CKG stands for Chongqing Jiangbei International Airport; AMS stands for Amsterdam International Airport. The number at the end of the duty refers to the number of sectors flown during the duty

As shown in Fig. 3, with the exclusion of the short period of low red alert due to sleep inertia, the simulation calculations show that the fatigue risk levels of the flight crews are similar for both policies going down, with a higher level of fatigue risk during the landing phase (as shown by the comparison between Fig. 3A and C), but the flight crew with the highest level of fatigue risk (the red alert period) is in a rest shift condition and is not during flight (as shown in Fig. 3A-f, C-p and Tables 5 and 6). A comparison of the results in Fig. 3B and D shows that the overall fatigue risk for flight crews on return trips under the exemption policy is higher than that under the CCAR-121 policy, but crew fatigue levels are lower in both cases and neither reaches the red alert level. Therefore, as observed from Fig. 3, the levels of fatigue risk for flight crews flying under the exemption policy during midday departures are either similar to the CCAR-121 policy (Fig. 3A and C) or higher than the CCAR-121 policy (Fig. 3B and D), but both are at a lower level of fatigue risk.

**Fig. 4:**
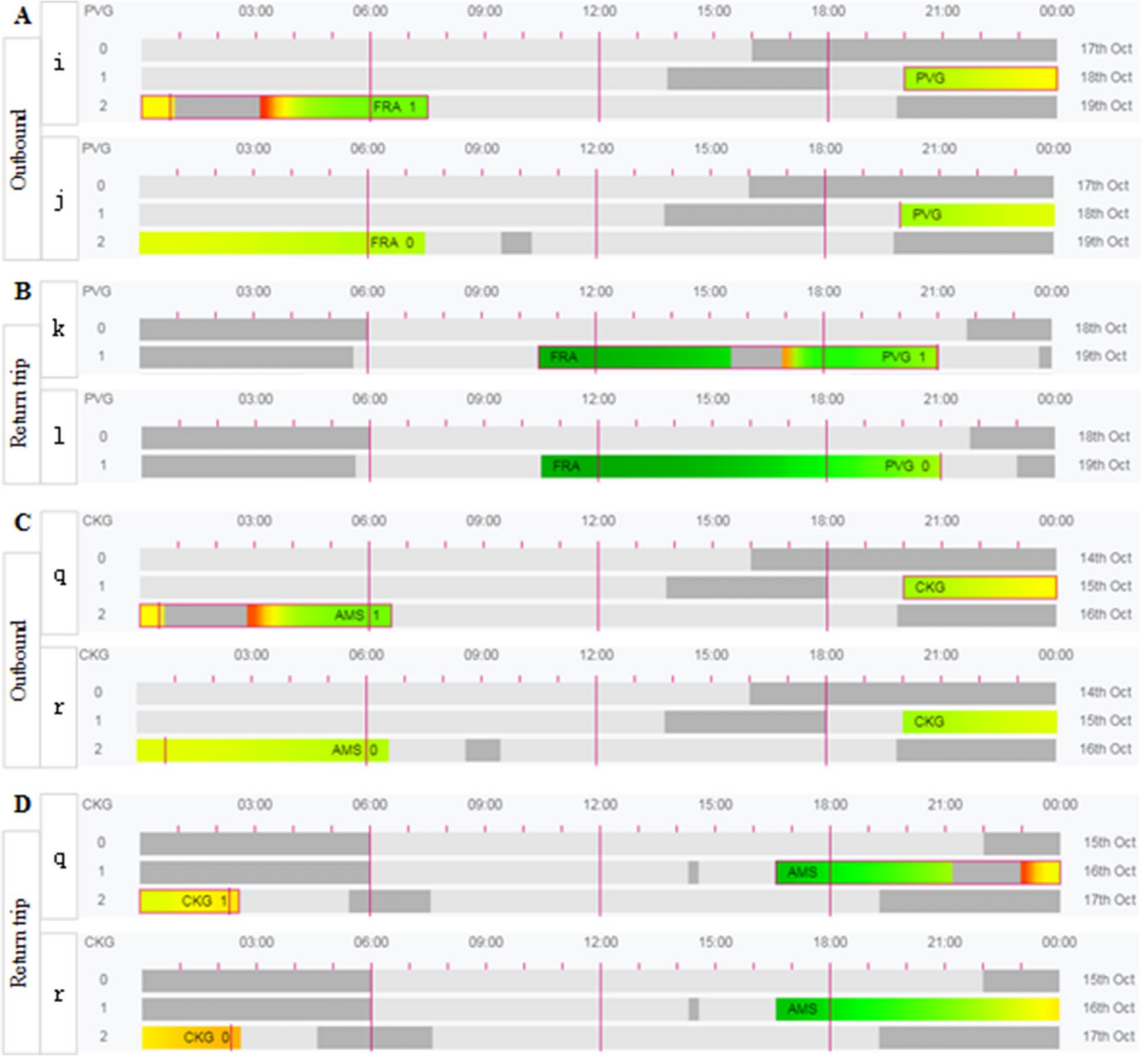
SAFE model simulation predictions for the 20:00 flight schedule under the exemption and the CCAR-121 Policies, with **A** and **B** showing the outbound (manned by flight crews i and j) and return (manned by flight crews k and l) flights, respectively, and **C** and **D** showing the outbound and return (both manned by flight crews q and r) flights, respectively, under the CCAR-121 policy. A comparison of **A** and **C** shows the fatigue risk for flight crews flying under the exemption approach and CCAR-121 regulations on outbound flights. A comparison of **B** and **D** shows a comparison of the fatigue risk levels for flight crews flying under the exemption approach and CCAR-121 regulations on return trip flights. Note: The meaning of the level of risk represented by the different colours is determined by Table 3. PVG stands for Shanghai Pudong International Airport; FRA stands for Frankfurt International Airport; CKG stands for Chongqing Jiangbei International Airport; AMS stands for Amsterdam International Airport. The number at the end of the duty refers to the number of segments flown during the time on duty

As shown in Fig. 4, with the exclusion of short periods of low red alert due to sleep inertia, the simulation calculations show that the fatigue risk levels for the flight crews are similar and low for both policies when going downrange, with the same level of risk and neither reaching the red alert level (as shown by the comparison between Fig. 4A and C). A comparison of the results in Fig. 4B and D shows that the overall fatigue risk level for flight crews on return trips under the exemption policy is lower than that under the CCAR-121 policy. Therefore, as observed from Fig. 4, the level of fatigue risk for flight crew members flying under the exemption policy is lower than that under the CCAR-121 policy for outbound crew members in the evening hours.

### Empirical results and comparison with model results

#### KSS

**Fig. 5:**
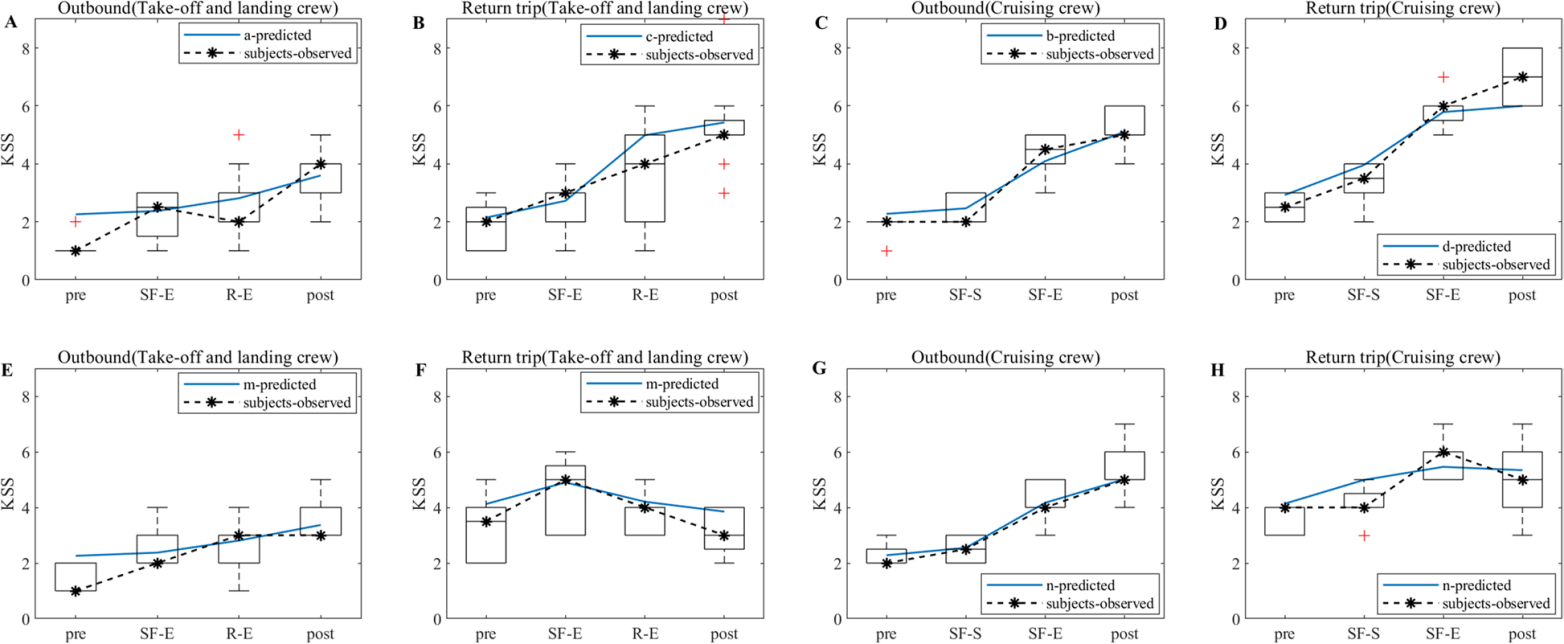
Comparison of flight crew fatigue risk model simulations and empirical KSS data (4 am)

**Fig. 6:**
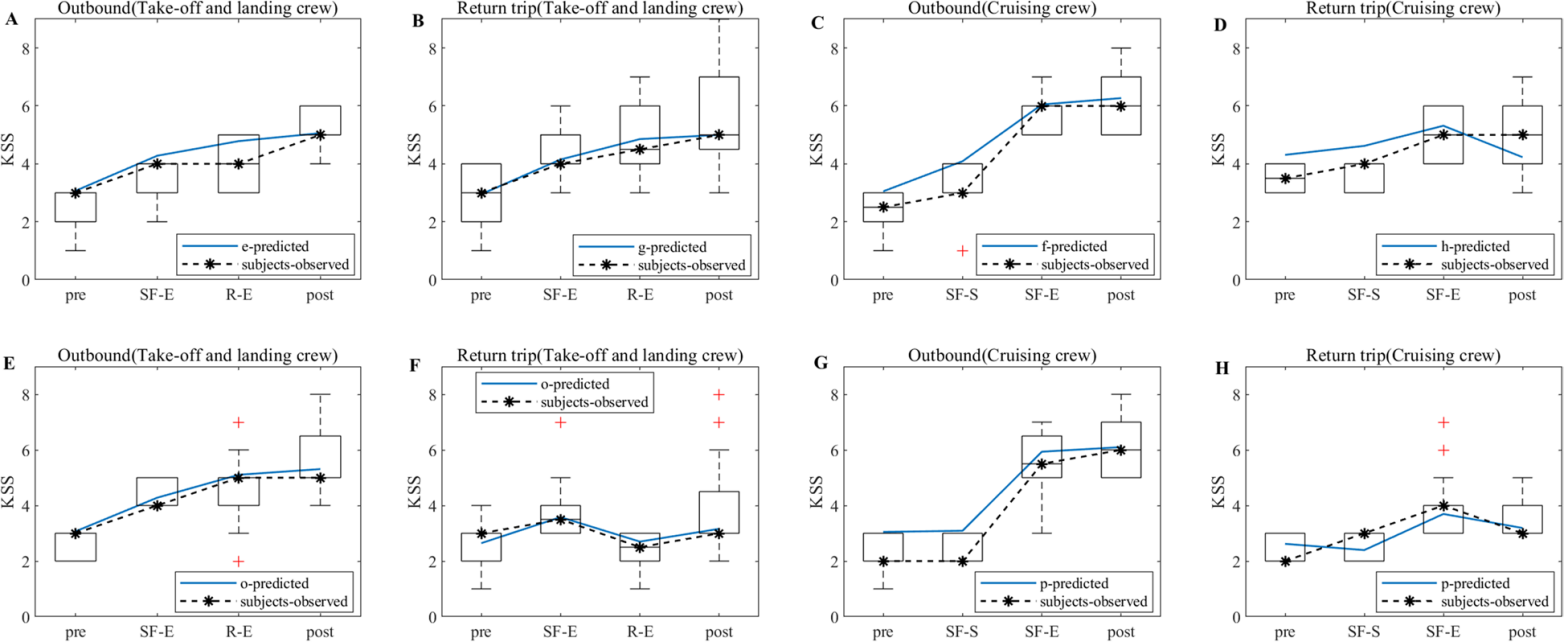
Comparison of flight crew fatigue risk model simulations and empirical KSS data (12:00 noon)

**Fig. 7:**
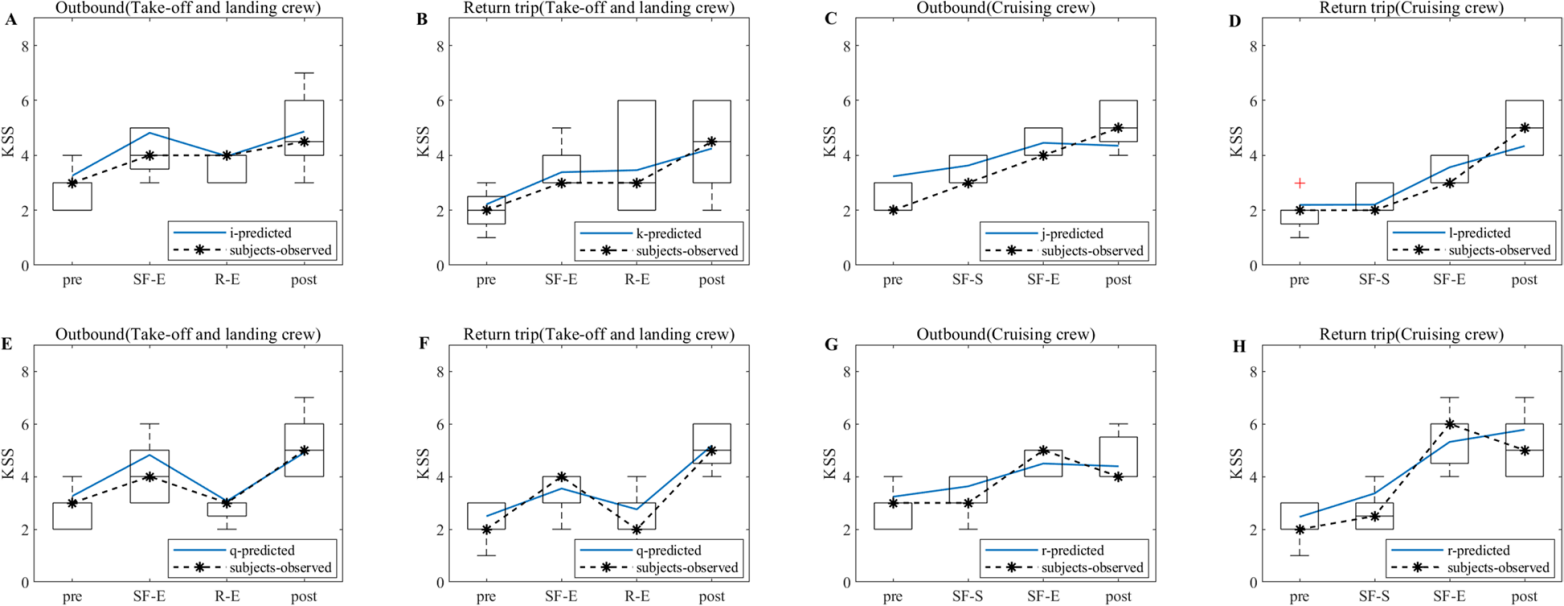
Comparison of flight crew fatigue risk model simulations and empirical KSS data (20:00)

According to the experimental flow shown in Fig. 1, the KSS data of the flight crew are collected from actual operation, and the KSS data derived from the model simulation are filtered according to the test nodes in Fig. 1, thus making a graph comparing the simulation results of the flight crew with the results of the empirical study, as shown in Figs. 5, 6 and 7. Note: In Figs. 5, 6 and 7, A and B show the simulation results of the KSS model for the take-off and landing crews of round-trip flights under the exemption policy compared with the results of the empirical study, respectively; C and D show the simulation results of the KSS model for the cruise crews of round-trip flights under the exemption policy compared with the results of the empirical study, respectively; E and F are the KSS model simulation results for the take-off and landing flight crews of round-trip flights under the CCAR-121 policy compared with the results of the empirical study, respectively; G and H are the KSS model simulation results for the cruise flight crews of round-trip flights under CCAR-121 policy compared with the results of the empirical study, respectively.

As observed from Fig. 5, first, the empirical KSS data and the model simulation KSS data results are holistically the same at the test nodes during flight, and the trends are nearly identical. The KSS scores for take-off and landing crews are then below 4 for both policies (Fig. 5A and E) and below 5 for the cruise group (Fig. 5C and G) during the outbound flight, indicating a lower level of fatigue risk for crews under both policies during the outbound flight. On the return flight, the take-off and landing crew KSS scores are below 5 for both policies (Fig. 5B and G), and the cruise crew KSS scores only appeared to be above 6 at the end of the flight for the flight crew under the exemption policy (Fig. 5D) but did not reach 6 until after the end of the cruise shift for both in-flight and CCAR-121 policy flights (Fig. 5D and H), indicating that cruise crew fatigue occurred at the end of the cruise flight for both policies.

As observed from Fig. 6, first, the empirical KSS data and the model simulation KSS data results also show the same overall consistency at the test nodes during flight, and the trends are nearly identical. The KSS scores of take-off and landing crews under the exemption policy then varied at flight nodes similar to those of CCAR-121 policy flight crews during the outbound flights, while the average KSS values during the flight varied less under the exemption policy than under the CCAR-121 policy (Fig. 6A and E). The cruise groups had a similar KSS score profiles under both policies, although a score of 6 was present (Fig. 6C and G). On the return flight, the KSS score for the take-off and landing groups, although higher than that of the CCAR-121 policy, remained below 4.5 during the flight (Fig. 6B and F), and the cruise group had the same situation as the take-off and landing groups (Fig. 6D and H).

As observed from Fig. 7, first, the empirical KSS data and the model simulation KSS data results also show the same overall consistency at the test nodes during flight, and the trends are nearly identical. Then, the KSS scores of the take-off and landing crews under the exemption policy varied at the flight node during the departing flight in a similar manner to those of CCAR-121 policy flight crews, both of which were below 4.5 (Fig. 7A and E); the KSS scores of cruise crews under the exemption policy are smaller than those under the CCAR-121 policy, both of which are again below 5 (Fig. 7C and G), indicating that both policies had lower levels of fatigue risk for crews on the departing flight. On the return trip of the flight, the KSS scores for the take-off and landing groups are smaller than those of the CCAR-121 policy, both of which are below 5 (Fig. 7B and F); the KSS scores for the cruise group under the exemption policy are below 5, while those under the CCAR-121 policy showed a score of 6 at the end of the cruise flight (Fig. 7D and H).

As observed from Figs. 5, 6 and 7, the KSS scores of the flight crews operating under the exemption policy show an increasing trend along the test nodes, i.e., the risk of flight crew fatigue increases with flight time, and therefore flight crews operating under the exemption policy need to perform fatigue mitigation at the end of their flight duties.

#### PVT

**Fig. 8:**
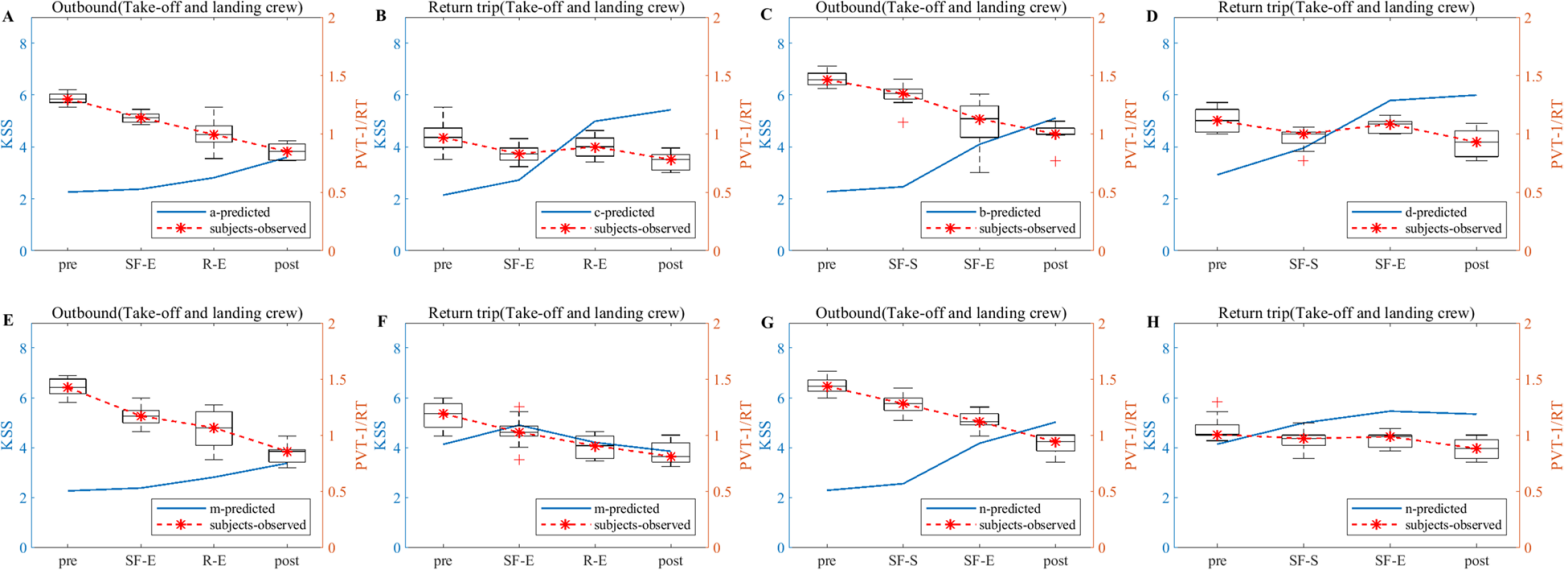
Comparison of flight crew fatigue risk model simulation and empirical PVT data (4 am)

**Fig. 9:**
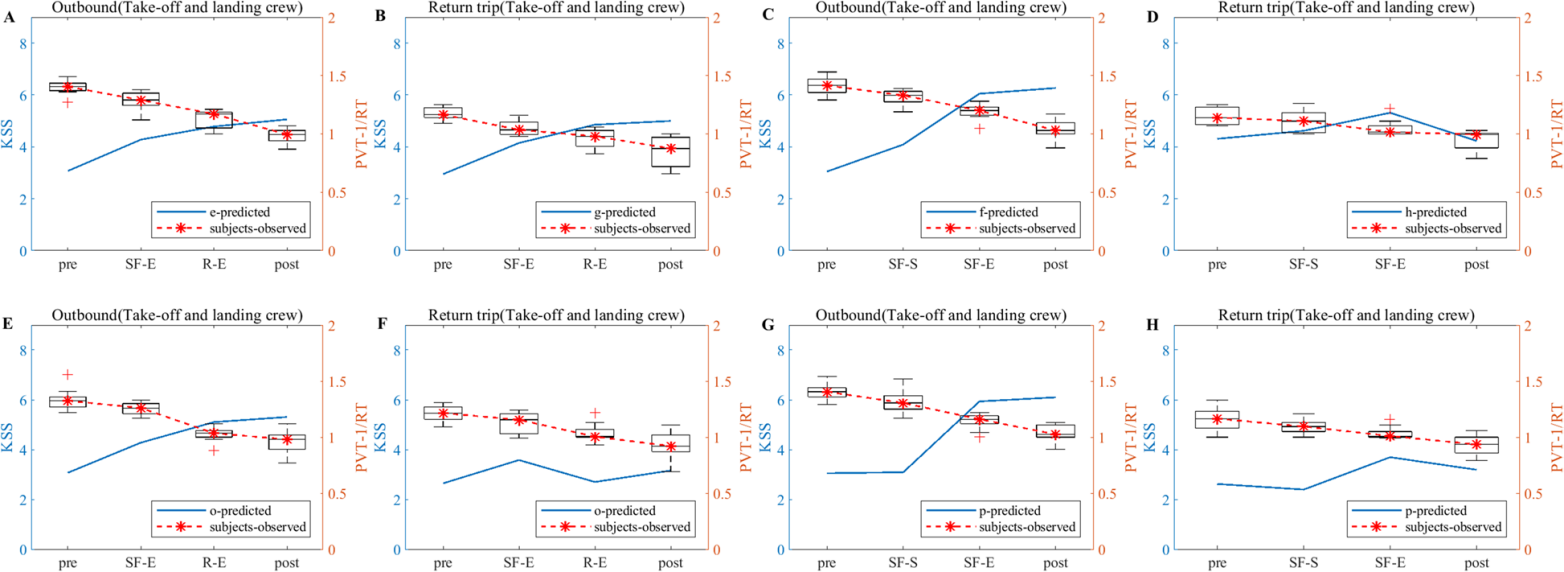
Comparison of flight crew fatigue risk model simulations and empirical PVT data (12:00 noon)

**Fig. 10:**
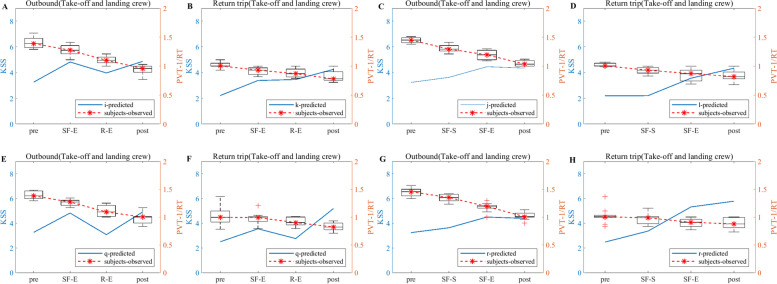
Comparison of flight crew fatigue risk model simulations and empirical PVT data (20:00)

Valid PVT data are screened for 48 pilots for the exemption approach and 60 pilots for the CCAR-121 policy. The screening criteria are based on criteria such as data at greater than a 3 s response time, no test time node data, and data at less than a 0.3 s response time. The KSS data derived from the model simulations are then combined to obtain a plot of the flight crew simulation results compared to the empirical study results, as shown in Figs. 8, 9 and 10. Note: A and B in Figs. 8, 9 and 10 show the PVT model simulation results for the take-off and landing crews of round-trip flights under the exemption policy compared with the results of the empirical study, respectively; C and D show the PVT model simulation results for the cruise crews of round-trip flights under the exemption policy compared with the results of the empirical study, respectively; E and F are the PVT model simulation results for the take-off and landing flight crews of the round-trip flights under the CCAR-121 policy compared with the results of the empirical study, respectively; G and H are the PVT model simulation results for the cruise flight crews of the round-trip flights under the CCAR-121 policy compared with the results of the empirical study, respectively.

As observed from Fig. 8, the overall PVT indicator data (1/RT) under both policies show a decrease with increasing flight duration, i.e., the longer the flight duration, the lower the level of alertness of the flight crew. At the same time, the magnitudes of the change in 1/RT of the crews under both policies are similar during the outbound phases of the flights (Fig. 8A and E indicate that the changes in 1/RT of the take-off and landing crews are between 0.7 and 1.5 under both policies; Fig. 8C and G indicate that the changes in 1/RT of the cruising crews are between 0.9 and 1.5 under both policies; the changes in 1/RT of the take-off and landing crews are greater than those of the cruising crews);On the return trips of the flights, the magnitude of flight crew 1/RT variation under the exemption policy is less than or similar to that under the CCAR-121 policy (Fig. 8B and F indicate that the magnitudes of take-off and landing crew 1/RT variations under the exemption policy are between 0.6 and 0.9, which are less than the magnitudes of take-off and landing crew 1/RT variations under the CCAR-121 policy (0.6–1.2); Fig. 8D and H indicate a range of change in cruise group 1/RT between 0.9 and 1.1 under both policies). In addition, with the exception of the CCAR-121 policy return take-off and landing groups (Fig. 8F), the vast majority of 1/RT data showed an inverse trend in flight time in relation to the KSS data.

As observed from Fig. 9, the PVT indicator data (1/RT) as a whole shows a decline with increasing flight duration, while the trend of the majority of 1/RT data on flight duration shows an inverse proportional relationship with KSS data, i.e., the longer the flight duration, the lower the level of alertness of the flight crew and the stronger the subjective fatigue. In addition, the magnitudes of the change in 1/RT for crews under the two policies are similar during the outbound phase of the flights (Fig. 9A and E indicate that the range of change in 1/RT for take-off and landing crews is between 1 and 1.5 under the two policies; Fig. 9C and G indicate that the range of change in 1/RT for cruising crews is between 1 and 1.5 under the two policies). The magnitudes of the 1/RT variation for the crews under both policies are also similar when the flight returns (Fig. 9B and F indicate a range of magnitude of the 1/RT variation for the take-off and landing crews between 0.9 and 1.2 under both policies; Fig. 9D and H indicate the range of magnitude of the 1/RT variation for the cruising crews is between 0.9 and 1.1 under both policies). It is also found that the magnitudes of 1/RT variation for the take-off and landing groups are greater than or similar to those of the cruise groups.

As observed from Fig. 10, the flight crew data situation for the 20:00 flight is similar to that of the 12 pm flight, i.e., the 1/RT data as a whole show a decline with increasing flight time, and the trend is inversely proportional to the KSS data. In addition, the magnitudes of the change in 1/RT of the crews are similar for both policies during the outbound flight (Fig. 10A and E indicate that the range of change in 1/RT of the take-off and landing crews is between 1 and 1.5 for both policies; Fig. 10C and G indicate that the range of change in 1/RT of the cruising crew is between 1 and 1.5 for both policies); The magnitudes of the change in 1/RT for the crews under both policies are also similar when the flight returned (Fig. 10B and F show that the changes in 1/RT for the take-off and landing crews are between 0.6 and 1 under both policies; Fig. 10D and H shows that the changes in 1/RT for the cruising crews are between 0.8 and 1 under both policies). Additionally, the magnitudes of 1/RT variation for the take-off and landing groups are greater than or similar to those of the cruise groups.

From the analysis of the KSS, which is a subjective scale of fatigue data, provided in Table 7, it was found that the average KSS value of the flight crew showed an exemption approach policy higher than Part CCAR-121 and breached the fatigue risk threshold (KSS value of 6) only during the early morning (4:00 am) departure, after the return cruise flight phase, but at this time and in subsequent phases, the flight crew was in a resting and nonworking condition and therefore had a low impact on flight safety. In other cases, the data for pilots operating under the exemption policy were overwhelmingly smaller than the data for pilots operating under the CCAR-121 policy, and the majority of average KSS values were below the fatigue risk threshold. Analysis of the PVT, which is an objective test for fatigue, in Table 8 shows that the average 1/RT values for pilots operating under the exemption approach were less than or similar to those of the CCAR-121 policy.

**Table 7:** Comparison of KSS data for flight crews operating under the two policies (Mean ± standard deviation $\overline{\mathrm{x}}$ ± s)

Moment of take-off	Flight phase	Operational policy and flight crew	Outbound				Return trip			
Early morning(4:00)	Take-off/landing	–	Pre	SF-E	R-E	Post	Pre	SF-E	R-E	Post
		Exemption policy (Group a flies the outbound trip; Group c flies the return trip)	1.20 ± 0.40	2.25 ± 0.83	2.60 ± 1.02	3.55 ± 0.74	1.90 ± 0.77	2.65 ± 0.73	3.75 ± 1.51	5.25 ± 1.30
		CCAR-121(Group m flies the round trip)	1.40 ± 0.49	2.45 ± 0.67	2.65 ± 1.01	3.65 ± 0.79	3.40 ± 1.11	4.50 ± 1.16	3.65 ± 0.65	3.05 ± 0.74
	Cruising	–	Pre	SF-S	SF-E	Post	Pre	SF-S	SF-E	Post
		Exemption policy (Group b flies the outbound trip; Group d flies the return trip)	1.80 ± 0.40	2.35 ± 0.48	4.45 ± 0.59	5.30 ± 0.649	2.50 ± 0.50	3.45 ± 0.59	5.90 ± 0.62	6.90 ± 0.83
		CCAR-121(Group n flies the round trip)	2.25 ± 0.43	2.50 ± 0.50	4.30 ± 0.64	5.35 ± 0.73	3.60 ± 0.49	4.05 ± 0.67	5.80 ± 0.68	4.95 ± 1.16
Noon(12:00)	Take-off/landing	–	Pre	SF-E	R-E	Post	Pre	SF-E	R-E	Post
		Exemption policy (Group e flies the outbound trip; Group g flies the return trip)	2.45 ± 0.67	3.60 ± 0.66	3.95 ± 0.86	5.25 ± 0.62	2.85 ± 0.96	4.25 ± 0.70	4.75 ± 1.18	5.65 ± 1.52
		CCAR-121(Group o flies the round trip)	2.55 ± 0.50	4.35 ± 0.48	4.80 ± 1.12	5.65 ± 1.11	2.45 ± 0.80	3.90 ± 1.34	2.40 ± 0.66	3.85 ± 1.53
	Cruising	–	Pre	SF-S	SF-E	Post	Pre	SF-S	SF-E	Post
		Exemption policy (Group f flies the outbound trip; Group h flies the return trip)	2.30 ± 0.78	3.35 ± 0.73	5.85 ± 0.73	6.05 ± 0.97	3.50 ± 0.50	3.65 ± 0.48	5.05 ± 0.86	4.95 ± 1.32
		CCAR-121(Group p flies the round trip)	2.25 ± 0.77	2.45 ± 0.50	5.50 ± 1.16	6.10 ± 1.09	2.45 ± 0.50	2.55 ± 0.50	3.90 ± 1.04	3.50 ± 0.59
Evening(20:00)	Take-off/landing	–	Pre	SF-E	R-E	Post	Pre	SF-E	R-E	Post
		Exemption policy (Group i flies the outbound trip; Group k flies the return trip)	2.70 ± 0.64	4.10 ± 0.77	3.60 ± 0.49	4.95 ± 1.20	2.00 ± 0.71	3.45 ± 0.59	3.90 ± 1.79	4.30 ± 1.49
		CCAR-121(Group q flies the round trip)	2.75 ± 0.54	4.15 ± 0.91	2.75 ± 0.43	5.00 ± 0.89	2.25 ± 0.77	3.55 ± 0.59	2.45 ± 0.92	5.10 ± 0.77
	Cruising	–	Pre	SF-S	SF-E	Post	Pre	SF-S	SF-E	Post
		Exemption policy (Group j flies the outbound trip; Group l flies the return trip)	2.45 ± 0.50	3.40 ± 0.49	4.40 ± 0.49	5.15 ± 0.80	1.90 ± 0.63	2.35 ± 0.48	3.40 ± 0.49	4.95 ± 0.86

**Table 8:** Comparison of PVT data for flight crews operating under the two policies (Mean ± standard deviation $\overline{\mathrm{x}}$ ± s)

Moment of take-off	Flight phase	Operational policy and flight crew	Outbound				Return trip			
Early morning(4:00)	Take-off/landing	–	Pre	SF-E	R-E	Post	Pre	SF-E	R-E	Post
		Exemption policy (Group a flies the outbound trip; Group c flies the return trip)	1.30 ± 0.05	1.14 ± 0.04	1.00 ± 0.12	0.85 ± 0.07	0.98 ± 0.13	0.83 ± 0.07	0.89 ± 0.09	0.77 ± 0.07
		CCAR-121(Group m flies the round trip)	1.43 ± 0.07	1.16 ± 0.08	1.05 ± 0.16	0.84 ± 0.09	1.18 ± 0.11	1.03 ± 0.13	0.90 ± 0.09	0.84 ± 0.10
	Cruising	–	Pre	SF-S	SF-E	Post	Pre	SF-S	SF-E	Post
		Exemption policy (Group b flies the outbound trip; Group d flies the return trip)	1.48 ± 0.06	1.33 ± 0.10	1.09 ± 0.20	1.00 ± 0.09	1.12 ± 0.10	0.96 ± 0.09	1.07 ± 0.05	0.92 ± 0.11
		CCAR-121(Group n flies the round trip)	1.44 ± 0.07	1.28 ± 0.08	1.13 ± 0.08	0.92 ± 0.08	1.06 ± 0.10	0.96 ± 0.08	0.96 ± 0.06	0.88 ± 0.08
Noon(12:00)	Take-off/landing	–	Pre	SF-E	R-E	Post	Pre	SF-E	R-E	Post
		Exemption policy (Group e flies the outbound trip; Group g flies the return trip)	1.40 ± 0.06	1.28 ± 0.08	1.13 ± 0.08	0.98 ± 0.06	1.18 ± 0.05	1.05 ± 0.06	0.96 ± 0.08	0.85 ± 0.12
		CCAR-121(Group o flies the round trip)	1.34 ± 0.09	1.25 ± 0.05	1.02 ± 0.06	0.96 ± 0.09	1.21 ± 0.07	1.13 ± 0.09	1.04 ± 0.08	0.92 ± 0.13
	Cruising	–	Pre	SF-S	SF-E	Post	Pre	SF-S	SF-E	Post
		Exemption policy (Group f flies the outbound trip; Group h flies the return trip)	1.41 ± 0.07	1.32 ± 0.06	1.19 ± 0.06	1.04 ± 0.08	1.15 ± 0.07	1.11 ± 0.09	1.05 ± 0.07	0.95 ± 0.08
		CCAR-121(Group p flies the round trip)	1.40 ± 0.07	1.31 ± 0.10	1.14 ± 0.07	1.03 ± 0.08	1.17 ± 0.11	1.10 ± 0.06	1.04 ± 0.05	0.93 ± 0.08
Evening(20:00)	Take-off/landing	–	Pre	SF-E	R-E	Post	Pre	SF-E	R-E	Post
		Exemption policy (Group i flies the outbound trip; Group k flies the return trip)	1.40 ± 0.10	1.28 ± 0.09	1.10 ± 0.06	0.94 ± 0.08	1.02 ± 0.05	0.92 ± 0.06	0.88 ± 0.07	0.83 ± 0.09
		CCAR-121(Group q flies the round trip)	1.39 ± 0.07	1.26 ± 0.05	1.11 ± 0.09	0.98 ± 0.10	1.01 ± 0.16	0.98 ± 0.10	0.91 ± 0.08	0.83 ± 0.07
	Cruising	–	Pre	SF-S	SF-E	Post	Pre	SF-S	SF-E	Post
		exemption policy (Group j flies the outbound, Group l flies the return trip)	1.45 ± 0.05	1.31 ± 0.06	1.20 ± 0.08	1.05 ± 0.05	1.02 ± 0.03	0.93 ± 0.06	0.85 ± 0.10	0.84 ± 0.09
		CCAR-121(Group r flies the round trip)	1.45 ± 0.07	1.34 ± 0.06	1.18 ± 0.08	1.02 ± 0.07	1.02 ± 0.14	0.97 ± 0.10	0.89 ± 0.077	0.88 ± 0.09

## Discussion

The exemption policy is a temporary deviation by the CAAC from the CCAR-121 policy on duty time and flight time for flight crews during the COVID-19 outbreak. As observed from Table 1, the maximum flight time under the exemption policy is 8–13 h more than that under the CCAR-121 policy, which increases the flight time, and it has been reported that the longer the flight time is, the greater the probability of an aviation accident. As Goode [32] found, the probability of a commercial aviation accident increases significantly with flight time, with 20% of US commercial aviation accidents occurring at 10 hours or more. In particular, note that the increase in flight time has led to an increase in flight cruising time and that such low workload flight periods can also cause symptoms of fatigue, such as low flight crew alertness [33] and the occurrence of microsleep conditions [34]. In addition, the number of flight crews under the exemption policy is twice that under the CCAR-121 policy, making the implementation of in-flight crew rest and rotation even more unavoidable in terms of avoiding flight time restrictions and relieving fatigue. In-flight rotational rest allows crew members to lie down and sleep during rest periods, and sleep on long flights is an operational countermeasure to fatigue and has been shown to improve subsequent alertness and performance, although resting in an in-flight rest facility is less rejuvenating than sleeping in a hotel or at home [35]. Therefore, based on the longer duty time, flight time, cruise time and more crew rotations under the above exemption policy compared to the CCAR-121 policy, a study on flight crew fatigue under the exemption policy is urgently needed. In addition, the exemption policy is a change from the CCAR-121 policy, so it is necessary to use the existing flight time limits in the CCAR-121 policy as a comparative benchmark to demonstrate the feasibility and scientific validity of the extended flight time provisions in the exemption policy.

Based on the biomathematical model’s characteristic of “comparing scores better than compliance thresholds” [6, 36] and the comparative results of the SAFE model assessment described above, it is concluded that flights departing in the early morning hours (4 am) and noon hours (12 pm) with crew fatigue levels operating under the exemption policy are either similar to or higher than those operating under the CCAR-121 policy but are higher than the phase portion of CCAR-121, either at a lower level of fatigue risk (Fig. 3B and D) or occurring during a shift break from flying duties (Fig. 2B-d and Table 5-d (10/19 3:00–5:00-"Resting “)). The level of crew fatigue risk for flights departing in the evening hours (20:00) when operating under the exemption policy is lower than the level of crew fatigue risk for flights operating under the CCAR-121 policy. As a result, the level of flight crew fatigue risk for flights operating under the exemption policy is similar to or lower than the level of flight crew fatigue risk for flights operating under the CCAR-121 policy, except for the high level of fatigue of the rotating rest crew during the return landing phase of early morning departures. It should be noted, however, that the CCAR-121 policy has been in operation for many years and its safety has been proven in actual operations. In summary, this paper applies the SAFE model to compare the model evaluation results under the two regulations and concludes that the overall fatigue risk levels of flight crews operating under the exemption policy are lower than those under the CCAR-121 policy, thus verifying the feasibility of the exemption approach and providing a solution for airlines to predict the fatigue risk levels of flight crews operating under the exemption approach.

In terms of empirical study validation, as observed from Figs. 5, 6 and 7, the results of the empirical KSS data analysis show that the empirical KSS data and the model simulation KSS data results are holistically the same at the test nodes during flight, and the trends are nearly identical. In addition, the KSS scores for flights departing in the early morning (4 am), noon (12 pm) and evening (20:00) hours are similar overall for both policies, while the KSS scores remained low during the flight, indicating that the fatigue risk levels are similar and at low levels for both policies, with only one 6-point average KSS score occurring at the end of the flight, especially for cruise crews; therefore, fatigue mitigation management after flight shifts should be strengthened, especially for cruise crews after their cruise flight shifts and at the end of the mission. In summary, the results of the empirical KSS data analysis show that the flight crew fatigue risk profiles are similar for the two policies, thus validating the similarity between the exemption and the CCAR-121 policies in terms of the subjective fatigue perception self-assessment method in terms of flight crew fatigue risk levels. As observed from Figs. 8, 9 and 10, the results of the empirical PVT data analysis show that for flights departing in the early morning (4 am), noon (12 pm) and evening (20 pm) hours, the magnitudes of change in 1/RT for flight crews under the exemption policy are less than or similar to those for crews under the CCAR-121 policy, regardless of whether the trips are outbound and return trips, again validating that the overall level of fatigue risk for flight crews operating under the exemption policy is lower than that under the CCAR-121 policy,. validating the feasibility of the exemption policy from the perspective of the objective alertness test methodology. In addition, with the exception of the return take-off and landing groups for flights departing in the early morning hours (Fig. 8F), the PVT indicator 1/RT data holistic showed an inverse proportional relationship with the trend of the model KSS data over the flight time. It is also found that flight crew alertness is less on the return trip of the flight than on the outbound trip and that the changes in alertness of the take-off and landing groups are greater or similar to that of the cruise crew, which is consistent with the findings of previous studies that alertness decreases with longer flight times and that the workload is higher during the take-off and landing phases than during the cruise phase [37–40].

At the same time, the model’s prediction results of flight crew fatigue are compared with the results of the empirical experimental study of real flight missions and are validated. The empirical KSS data are basically consistent with the model KSS data, and the PVT empirical results also showed an inverse proportional relationship with the model KSS analysis results overall. Therefore, the empirical study not only verifies the feasibility of the exemption policy but also validates the accuracy and application value of the SAFE model in predicting crew fatigue conditions by comparing it with the model simulation results.

In terms of theoretical analysis and practical demonstration of the two policies, it can be observed from Table 1 that when 3 sets of crews are set to fly round-trip flights to meet the requirements of the exemption policy, the 3 sets of crews (6 pilots) can be regarded as 2 sets of expanded flight crews with 3 pilots under the CCAR-121 policy; in terms of flight time, the total flight time under the exemption policy is divided into 13 hours or 11.5 hours each way, while the flight time under the exemption policy is less than or equal to the flight time under the CCAR-121 policy, as an expanded flight crew with 3 pilots is required to fly 13 hours each way according to CCAR 121. When 4 sets of crews are set to fly round-trip flights to meet the requirements of the exemption policy, the 4 sets of crews (8 pilots) can be considered as 2 sets of augmented flight crews with 4 pilots under the CCAR-121 policy. In terms of flight time, the total round trip flight time under the exemption policy is divided into 15 hours or 11.5 hours each way, whereas under CCAR 121, the flight time for an expanded flight crew with 3 pilots is 17 hours each way; therefore, the flight time under the exemption policy is also less than or equal to that of the CCAR-121 policy. In summary, the average flight time per pilot under the exemption policy is less than that under the CCAR-121 policy, and the duty time is similar. In addition, since the issuance of the exemption policy, the airlines concerned have operated safely for more than 10,000 hours/thousands of flights without a single unsafe incident, providing a favourable practical validation of the exemption policy at the operational level. At the same time, for flights operated under the exemption policy, 3/4 sets of crews are used, and the crews are provided with a resting place on board to meet the requirements. The crews return without spending the night at the destination, thus allowing the pilots to work and rest according to the base time throughout the mission and reducing fatigue caused by jet lag.

### Limitations

The assessment of flight crew fatigue under different departure times was developed only from 04:00, 12:00 and 20:00, but its representation of the early morning, noon and evening flight departure time periods is not comprehensive. In addition, only the flight crew fatigue assessment study has been conducted for only the 20–22-hour flight time limit, while the flight crew fatigue assessment for exempted flights above 22 hours in the exemption policy needs to be further studied. This study is also limited to international flights with a 7–8 time zone difference, while the assessment of flight crew fatigue for other larger time differences also needs further study.

### Strengths

This is the first study to analyse the assessment of pilot fatigue on international flights operated under the new exemption policy of the Civil Aviation Administration of China during the COVID-19 epidemic, while also providing data support for the analysis of the current situation of pilot fatigue on international flights. In addition, by comparing the fatigue of pilots on flights under the CCAR-121 policy, it was verified for the first time that the fatigue levels of pilots on flights operating under the exemption policy were at safe levels. Flight crew fatigue assessments under both policies are carried out in a comprehensive manner based on the following three fatigue assessment methods: biomathematical models, subjective scales and objective tests; thus, the research methods are more comprehensive. In addition, pilot fatigue was analysed for flights departing at different times of the day, and data and references can be provided to study the impact of different departure moments on the risk of pilot fatigue.

## Conclusion

Crew onboard rotations are complex, especially with the current international flight exemptions where multiple sets of 6–8 crew members are on board at the same time, and ensuring the coordination of crew rotations in this mode largely determines flight safety. Therefore, the results of predicting and monitoring the fatigue risk of flight crews during duty can inform the design and adjustment of in-flight rotation plans under the exemption scheme regulations. Based on the SAFE model, this paper simulates flight plans under both the exemption and CCAR-121 policies and verifies that the level of crew fatigue risk under the exemption is similar to that under CCAR-121 and that the overall risk is lower. This paper also conducts an empirical test on the fatigue of flight crews operating under the two policies based on the KSS subjective fatigue self-assessment scale and the PVT objective alertness test from both subjective and objective perspectives. In addition, based on the fact that the CCAR-121 policy has been operating safely for many years and its safety has been proven numerous times in actual operations and based on the above results, it is concluded that it is equally safe and feasible for airlines to implement an exemption policy during the COVID-19 outbreak.

## Data Availability

The datasets generated and/or analysed during the current study are not publicly available due to the commercial airline protocol signed with Chinese airlines. However, they are available from the corresponding author upon reasonable request.
